# Crystal Structure of a Novel *N*-Substituted L-Amino Acid Dioxygenase from *Burkholderia ambifaria* AMMD

**DOI:** 10.1371/journal.pone.0063996

**Published:** 2013-05-28

**Authors:** Hui-Min Qin, Takuya Miyakawa, Min Ze Jia, Akira Nakamura, Jun Ohtsuka, You-Lin Xue, Takashi Kawashima, Takuya Kasahara, Makoto Hibi, Jun Ogawa, Masaru Tanokura

**Affiliations:** 1 Department of Applied Biological Chemistry, Graduate School of Agricultural and Life Sciences, The University of Tokyo, Tokyo, Japan; 2 Institute of Biophysics, Chinese Academy of Sciences, Beijing, China; 3 Division of Applied Life Sciences, Graduate School of Agriculture, Kyoto University, Kyoto, Japan; 4 Laboratory of Industrial Microbiology, Graduate School of Agriculture, Kyoto University, Kyoto, Japan; University of Queensland, Australia

## Abstract

A novel dioxygenase from *Burkholderia ambifaria* AMMD (SadA) stereoselectively catalyzes the C3-hydroxylation of *N*-substituted branched-chain or aromatic L-amino acids, especially *N*-succinyl-L-leucine, coupled with the conversion of α-ketoglutarate to succinate and CO_2_. To elucidate the structural basis of the substrate specificity and stereoselective hydroxylation, we determined the crystal structures of the SadA.Zn(II) and SadA.Zn(II).α-KG complexes at 1.77 Å and 1.98 Å resolutions, respectively. SadA adopted a double-stranded β-helix fold at the core of the structure. In addition, an HXD/EX_n_H motif in the active site coordinated a Zn(II) as a substitute for Fe(II). The α-KG molecule also coordinated Zn(II) in a bidentate manner via its 1-carboxylate and 2-oxo groups. Based on the SadA.Zn(II).α-KG structure and mutation analyses, we constructed substrate-binding models with *N*-succinyl-L-leucine and *N*-succinyl-L-phenylalanine, which provided new insight into the substrate specificity. The results will be useful for the rational design of SadA variants aimed at the recognition of various *N*-succinyl L-amino acids.

## Introduction

The hydroxy amino acids, which are components of glycopeptide antibiotics, cyclodepsipeptides and collagen, have many physiological activities. Some hydroxy amino acids can also be used as precursors in the asymmetric synthesis of pharmaceuticals [1]. For example, (2*S,*3*R,*4*S*)-4-hydroxyisoleucine has insulinotropic and anti-obesity effects and seems to have potential for the treatment of diabetes [2]. In addition, *cis*-4-hydroxy-L-proline has been clinically evaluated as an anticancer drug [3].

The hydroxylation of amino acids is catalyzed by the ferrous [Fe(II)]- and α-ketoglutarate (α-KG)-dependent dioxygenases. These enzymes can also hydroxylate proteins, nucleic acids, lipids and small molecules [4], [5]. They participate in a vast array of protein side-chain modifications, repair of alkylated DNA/RNA, and biosynthesis of antibiotics and plant products [6]. Dioxygenase-mediated hydroxylation requires dioxygen as well as Fe(II) and α-KG. One of the oxygen atoms is incorporated into the substrate to form hydroxy amino acid, while the other oxygen atom is used to oxidatively break down α-KG into succinate and CO_2_. This family of enzymes possesses a common protein fold, which is called the double-stranded β-helix (DSBH) fold, as the core of the structure, and an HXD/EXnH motif in the active site to coordinate the Fe(II) cofactor [7]–[9]. The α-KG binding sites are relatively conserved and α-KG binds to the iron in a bidentate manner via its 1-carboxylate and 2-oxo groups. However, there is much more variation in the secondary substrate-binding sites, which defines the substrate specificity and stereoselectivity of the hydroxylation reaction.

**Figure 1:**
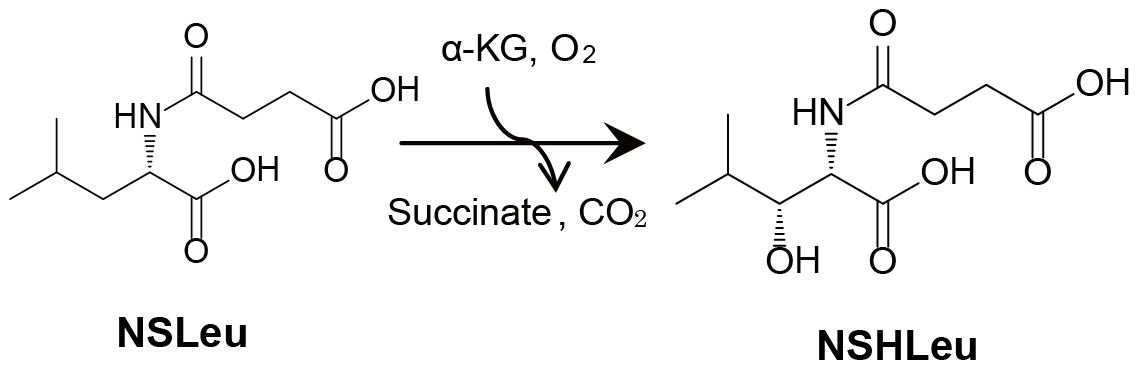
Enzymatic reaction scheme for the SadA-mediated hydroxylation of NSLeu.

SadA is a member of the dioxygenase family from *Burkholderia ambifaria* AMMD. This enzyme is useful as a novel biocatalyst for the (*R*)-selective hydroxylation at the C-3 position of *N*-substituted branched-chain L-amino acids, especially *N*-succinyl-L-leucine (NSLeu), to produce *N*-succinyl-(2*S*,3*R*)-3-hydroxyleucine (NSHLeu) with >99% stereoselectivity (Fig. 1) [10]. (2*S*,3*R*)-3-hydroxyleucine is a promising material for the preparation of certain cyclic depsipeptides which function as platelet aggregation inhibitors and is also a component of the antibiotic lysobactin [11], [12]. In a preceding study [10], *N*-formyl-L-leucine, *N*-acetyl L-leucine and *N*-carbamyl-L-leucine were also recognized as substrates by SadA, whereas the activities toward them were low (2–22%) compared with that toward NSLeu. SadA showed almost the same activity for several kinds of *N*-substituted branched-chain L-amino acids, *N*-succinyl-L-valine, *N*-succinyl-L-isoleucine and NSLeu (data not shown). In addition, SadA is the first characterized Fe(II)- and α-KG-dependent dioxygenase that catalyzes *N*-substituted aromatic L-amino acids, especially *N*-succinyl-L-phenylalanine (NSPhe), although its activity toward NSPhe is lower than that toward NSLeu (data not shown). Thus, SadA has the potential for widely producing C3-hydroxylated amino acids with various types of branched chain or aromatic ring.

Sequence alignment shows that SadA shares at most 12% sequence identity with other family members (PHD2, PDB ID 3HQR), which gives us only the Fe(II)-binding HXD/EXnH motif as an enzymatic property. Although a few Fe(II)/α-KG-dependent dioxygenases are known to hydroxylate free amino acids [13], [14], their substrate specificities are restricted to hydrophilic amino acids such as L-arginine and L-asparagine. Therefore, the mechanism for the substrate specificity of SadA remains poorly understood. Here we report the structures of SadA.Zn(II) and SadA.Zn(II).α-KG. In addition, based on the structures and mutation analyses, we propose a substrate-binding model to elucidate the structural basis of the substrate specificity and stereoselective hydroxylation.

## Materials and Methods

### Protein Purification and Crystallization

The *Escherichia coli* Rosetta(DE3) cells (Novagen) harboring the pQE80 vector (QIAGEN) with full-length SadA gene were grown in Lysogeny Broth medium and incubated at 310 K until the OD_600_ reached 0.6–0.8. Isopropyl β-D-1-thiogalactopyranoside (IPTG) was added at a final concentration of 0.5 mM and the culture was then further incubated at 298 K overnight. After harvesting, the cells were disrupted by sonication in the resuspending buffer [20 mM Tris-HCl (pH 8.0), 10 mM imidazole, 0.5 M NaCl and 1 mM dithiothreitol (DTT)] and the cell debris was removed by centrifugation. SadA was trapped on Ni-NTA Superflow resin (QIAGEN). After washing, the protein was eluted and further purified by using Resource Q (GE Healthcare). The solution containing purified SadA was concentrated to 15 mg ml^−1^ in 20 mM Tris-HCl (pH 8.0), 1 mM DTT for crystallization.

SadA was crystallized using the sitting-drop vapor diffusion method without any metals such as Zn(II) added to the solution during purification and crystallization. The crystals were obtained by mixing 1.0 µl protein solution with 1.0 µl reservoir solution consisting of 0.1 M CHES (pH 9.5) and 30% (w/v) PEG 3,000 at 293 K. The purification and crystallization of selenomethionine-substituted SadA (SadA^SeMet^) were performed as reported previously [15]. The cosubstrate α-KG was added to the protein solution to a final concentration of 10 mM and was cocrystallized with SadA seed crystals under the same crystallization conditions.

### Data Collection and Processing

The X-ray diffraction data of SadA.Zn(II) and SadA.Zn(II).α-KG complex crystals were collected on the AR-NW12A and AR-NE3A beamlines at Photon Factory (Tsukuba, Japan), respectively. For phasing by single-wavelength anomalous dispersion (SAD) of selenium atoms, we collected the X-ray diffraction data of SadA^SeMet^ on the BL-17A at Photon Factory. All diffraction data were indexed, integrated, and scaled with the program *HKL*-2000 [16]. The data-collection and processing statistics are summarized in Table 1.

**Table 1:** Data-collection statistics.

	SadA^SeMet^.Zn(II)	SadA.Zn(II)	SadA.Zn(II).α-KG	
**Data Collection**				
Beamline	PF BL-17A	PF AR-NE3A		PF AR-NW12A
Wavelength (Å)	0.97894	1.00000		1.00000
Space group	*P*2_1_2_1_2_1_	*P*2_1_2_1_2_1_		*P*2_1_2_1_2_1_
	*a* = 49.3,	*a* = 49.0,		*a* = 49.5,
Unit-cell parameters (Å)	*b* = 70.9,	*b* = 71.0,		*b* = 71.1,
	*c* = 148.2	*c = *147.9		*c = *147.9
Resolution (Å)	20.0−2.40	50.0−1.77		50.0−1.98
	(2.44−2.40)	(1.80−1.77)		(2.01−1.98)
No. of unique reflections	20990 (1041)	51288 (2448)		37009 (1808)
Redundancy	14.3 (14.2)	13.8 (10.5)		7.1 (6.5)
Completeness (%)	100 (100)	99.6 (97.3)		98.9 (100.0)
*R*_sym_^a^	0.087 (0.423)	0.066 (0.317)		0.056 (0.252)
<*I*/σ(*I*)>	64.2 (7.8)	68.2 (9.5)		56.3 (9.5)
**Phasing**	SAD^b^			
Figure of merit after phasing	0.37			
Figure of merit after density modification	0.88			
**Refinement**				
*R*_work_/*R*_free_(%)		21.1/24.1	21.2/27.8	
r.m.s.d.^c^				
Bond length (Å)		0.021	0.018	
Bond angle (°)		2.17	2.21	
No. of atoms				
Protein		3959	3956	
Ligand/ion		2	12	
Water		125	138	
Average *B*-factor (Å^2^)				
Protein		25.6	41.7	
Ligand/ion		34.6	58.2	
Water		22.6	38.1	
Ramachandran analysis^d^				
Favored, allowed, outliers (%)		99.0, 1.0, 0	98.2, 1.6, 0.2	

Crystal parameters and data collection and refinement statistics.

Values in parentheses are for the highest resolution shell.

a where *I_i_* is the *i*th intensity measurement of the reflection *hkl*, including symmetry-related reflections, and is its average.

b Single-wavelength anomalous dispersion.

c r.m.s.d., root mean square deviation.

d Calculated by MolProbity.

### Structure Analysis

The initial phase of SadA^SeMet^ was obtained by SAD using the CNS program suite [17]. Seventeen of the twenty selenium atoms in the asymmetric unit were identified. After selenium atom search and phase calculations, the model building was automatically carried out with BUCCANEER [18]. Manual rebuilding and refinement were performed with COOT [19] and REFMAC5 [20] from the CCP4i program suite, respectively. The structures of SadA.Zn(II) and SadA.Zn(II).α-KG were determined by the molecular replacement method with the MOLREP program [21] using the SadA^SeMet^ structure as the initial model. Manual rebuilding and refinement were performed with COOT and REFMAC5, respectively. The data-collection and processing statistics are summarized in Table 1.

### Ligand Docking Simulations

The initial model of SadA.Zn(II).α-KG.NSLeu was constructed using the Molecule Builder of the molecular operating environment (MOE; Chemical Computing Group, Montreal, Canada). The initial model was minimized by employing the Merck Molecular Force Field 94x (MMFF94x). The substrate-binding site of the SadA structure was detected using the Alpha Site Finder in MOE. For NSLeu, 250 conformations were generated using the default LowModeMD search parameters. Ligand docking simulations were performed using the ASEDock program of MOE. The molecular dynamics (MD) simulations were performed using MMFF94x with the Nose-Poincare-Anderson (NPA) algorithm and the generalized Born method. The distance and relative position between Zn(II) and C3 of NSLeu were fixed to 3.0–3.5 Å [13]. MD minimization was run with a time step of 0.001 ps until the model energy was converged. *N*-succinyl-L-phenylalanine (NSPhe) was also docked in the structure of the SadA.Zn(II).α-KG complex in the same way. The final model was obtained by perturbing the MD minimized model based on activity analyses.

### Construction of Expression Plasmids of SadA Mutants

Site-directed mutageneses were performed by PCR with a QuikChange kit (Stratagene, La Jolla, CA) and pQE80-SadA plasmid as a template [22]. The primers for mutants are summarized in Table S1. The mutations were confirmed by DNA sequencing. SadA mutants were expressed and purified according to the method described for wild-type SadA.

### Activity Assay

The activity assay was performed as described previously [10]. In a preceding study, it was confirmed that Fe(II) functions as an active cofactor for SadA but Zn(II) does not (data not shown). Briefly, a reaction mixture composed of 10 mM substrate, 15 mM α-KG, 0.5 mM FeSO_4_·7 H_2_O, 10 mM L-ascorbate, 50 mM Tris-HCl buffer (pH 8.0), and 1 mg ml^−1^ purified SadA was used. The reaction was allowed to proceed at 30°C for 2 h and was terminated by the addition of 20 mM EDTA. The *N*-succinyl group of the products was desuccinylated by adding a 1/50 vol of 6 M HCl to the reacted solution and heating at 105°C for 1 h. After neutralization with NaOH, the hydroxy amino acids in the reaction mixture were derivatized with AccQ Tag (Waters, Milford, MA) according to the manufacturer’s instructions. The derivatives were analyzed using an Alliance 2695 high-performance liquid chromatography (HPLC) system (Waters) equipped with a fluorescence detector. An XBridge C_18_ column (5 µm; 2.1 mm×150 mm; Waters) was used for separation at 40°C. All measurements were performed in triplicate.

### Metal Analysis

The wild-type enzyme was expressed and purified as described above. Protein concentrations were about 23 mg ml^−1^ and the metal content of the enzyme was determined by inductively coupled plasma atomic emission spectrophotometer (ICP-AES).

## Results

### Overall Structures of SadA Complexes

**Figure 2:**
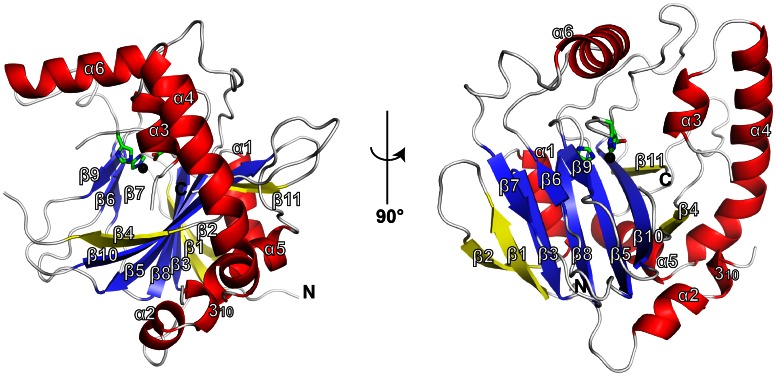
Ribbon representation of the SadA overall structure. The core β-strands of the DSBH fold and the surrounding β-strands are shown in deep blue and yellow, respectively. α-helices are shown in red. Metal-binding residues are represented by green sticks. Zn(II) is displayed as a black sphere (substituting for iron at the active site).

The crystal structures of SadA.Zn(II) and SadA.Zn(II).α-KG were determined at 1.77 Å and 1.98 Å resolutions, respectively. The electron density maps of residues Ser60–Thr74 and Ala148–Phe152 were not observed in either of the structures. The structure of SadA.Zn(II) contained 11 β-strands, 6 α-helices and one 3_10_ helix, and possessed the DSBH fold at its core (Fig. 2), which was adopted in most of the α-KG-dependent dioxygenases [8], [23], [24]. The DSBH fold of SadA was comprised of seven β-strands, four of which (β3, β5, β8 and β10) formed a major β-sheet and the other three of which (β6, β7 and β9) constituted a minor β-sheet. The β1, β2, β4 and β11 strands extended the major β-sheet. Six α-helices (α1-α6) were packed along the major β-sheet of the DSBH fold.

**Figure 3:**
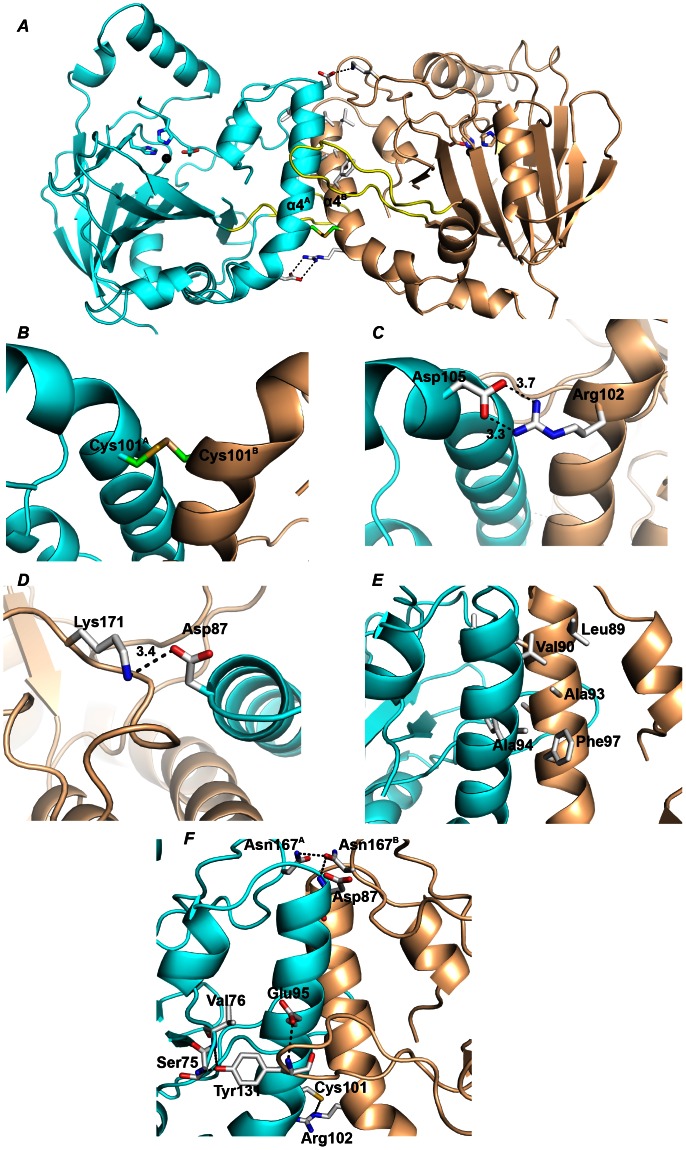
Dimer assembly of SadA. ***A***, Ribbon representation of the SadA dimer in an asymmetric unit. The two protomers A and B are colored cyan and wheat, respectively. The loop between α5 and β4 is colored yellow. ***B***, The disulfide linkage is shown as a stick model in the dimeric structure. The salt bridges of Asp105-Arg102 (***C***) and Lys171-Asp87 (***D***), the hydrophobic amino acids (***E***) and the hydrogen bonds (***F***) are shown as white sticks. Distances are given in angstroms.

SadA formed a dimer in the crystals as well as in solution (Fig. 3*A* and Fig. S1). The dimeric contact area is mainly comprised of the residues of α4 and the loop between α5 and β4. The dimer forms an intermolecular disulfide bond of Cys101^A^-Cys101^B^ and two salt bridges of Lys171–Asp87 (3.4 Å) and Asp105–Arg102 (3.2 and 3.7 Å) (Fig. 3*B*–*D*). The hydrophobic interactions are formed by the side chains of Leu89, Val90, Ala93, Ala94 and Phe97 (Fig. 3*E*). Moreover, two protomers form several intermolecular hydrogen bonds, i.e. Ser75 N–Tyr131 OH (2.4 Å), Asp87 N–Asn167 OD1 (3.7 Å), Arg102 N–Cys101 SG (3.8 Å), Tyr131 N–Glu95 OE2 (3.4 Å), Tyr131 OH–Val76 N (3.7 Å) and Asn167 OD1–Asn167 ND2 (3.6 Å) (Fig. 3*F*). These interactions serve as key structural features in stabilizing the dimer formation, and the dimer interface was calculated to have a buried surface area of 1,131 Å^2^ per protomer by the PISA server [25]. The dimers of SadA.Zn(II) and SadA.Zn(II).α-KG are structurally identical within 0.17 Å r.m.s.d. for 444 C_α_ atoms.

### Characteristics of the Active Site

In the SadA.Zn(II).α-KG structure, the active site is surrounded by the loop of β4-β5 and the β9 strand. The structure possesses a conserved HXD/EX_n_H motif. The electron density map of metals can be observed in the active site. We have performed crystallization and soaking experiments with Fe(II) under aerobic or anaerobic conditions, but failed to obtain the crystal with Fe(II). The data from inductively coupled plasma atomic emission spectroscopy (ICP-AES) showed that the concentration of Zn(II) was about 14-fold higher than that of Fe(II) in the SadA solution (Table S2); therefore, the metal was modeled as Zn(II) substituting for Fe(II). Zn(II) is coordinated by the side chains of His155, Asp157 and His246, which are conserved in the dioxygenase superfamily [7], [22], [23].

**Figure 4:**
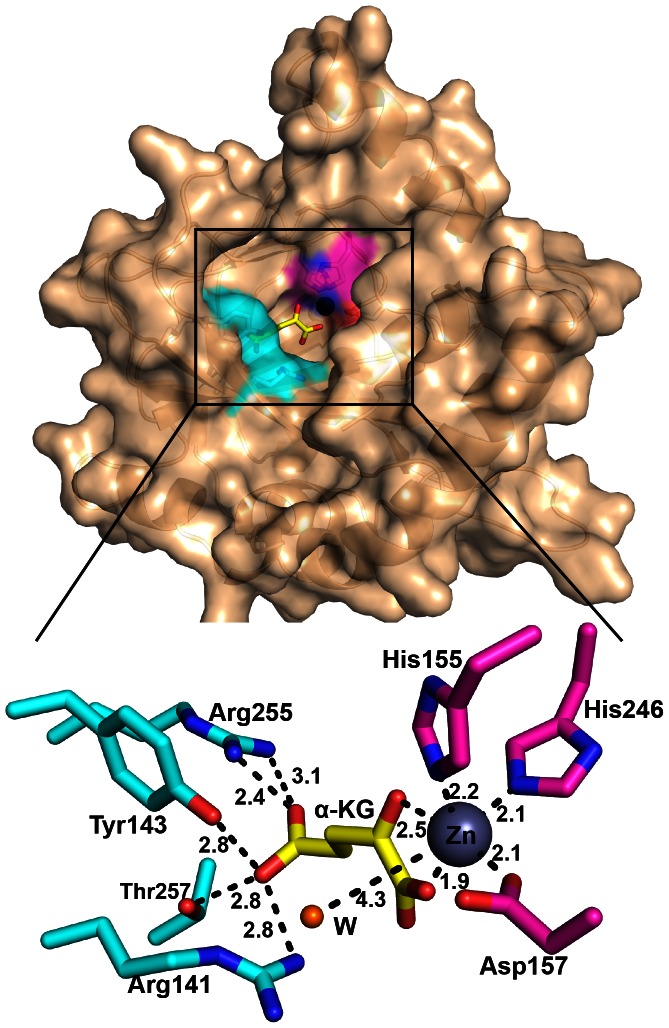
Overview of the active site in the SadA.Zn(II).α-KG structure. The surface is colored wheat. Zn(II) is shown as a deep blue sphere that is coordinated by three SadA residues (shown as the magenta surface region and magenta sticks), a water (orange sphere), and α-KG (yellow sticks). The residues which bind to C5 of α-KG are colored cyan. Black dashes indicate metal coordination and selected hydrogen bonds.

On the other hand, only one α-KG molecule is clearly observed in chain A of the SadA.Zn(II).α-KG structure (Fig. S2). The α-KG coordinates Zn(II) in a bidentate manner using its 2-oxo carbonyl and C-1 carboxylate groups, which form an octahedral coordination geometry complex (Fig. 4). The 2-oxo oxygen of α-KG is located trans to Asp157 and the C-1 carboxylate is observed to be trans to His155 of the HXD/EXnH motif. The C-5 carboxylate forms three salt bridges with the side chains of Arg141 (2.8 Å) and Arg255 (2.4 Å, 3.1 Å), and two hydrogen bonds with the hydroxy group of Tyr143 (2.8 Å) and Thr257 (2.8 Å). A single water molecule is observed to be trans to His246 of the HXD/EX_n_H motif. This water would be displaced by O_2_ in the course of the catalytic reaction.

### Substrate Recognition and Specificity

We have performed cocrystallization and soaking experiments with *N*-oxalylglycine (NOG, an α-KG analogue) and NSLeu under aerobic or anaerobic conditions, but failed to obtain the complex structure. The SadA.Zn(II).α-KG structure has a deep cavity that is large enough to accommodate the substrate (Fig. S3). By comparing the complex structures of the family enzymes with their substrates [7], [13], [23], [26], we found that the active-site residues and the bound zinc ion are conserved, which suggested that the SadA.Zn(II).α-KG structure is in a state capable of accepting a substrate.

**Figure 5:**
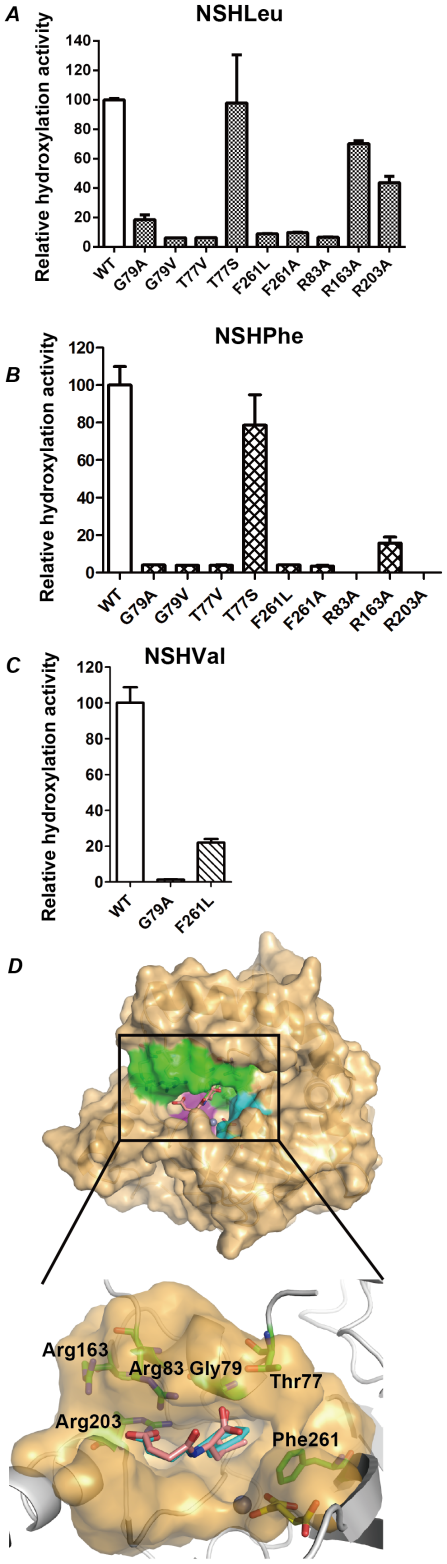
Structural basis of SadA.Zn(II).α-KG.NSLeu and SadA.Zn(II).α-KG.NSPhe. The relative hydroxylation activity of SadA mutants were measured toward NSLeu (***A***) and NSPhe (***B***). The activities of wild-type SadA are represented as 100 and error bars are standard deviations. ***C***, NSLeu (salmon) and NSPhe (cyan) were docked into the SadA.Zn(II).α-KG structure. Zn(II) is shown as a deep blue sphere and α-KG is shown as yellow sticks. The predicted residues which bind the substrate are shown as the green surface region and green sticks.

Based on these observations, we attempted to build the docking model with NSLeu. Initially, the MOE suite was used to predict the locations of the NSLeu molecule in the active site, and we presumed the presence of several residues related to substrate-binding of SadA, including Arg83, Arg163 and Arg203, which may form an electropositive-rich cavity. Gly79 and Phe261 may undergo a hydrophobic interaction in the course of substrate recognition (Fig. S3). The results of the mutation analyses of the predicted residues to evaluate whether the mutations affect the SadA activity toward NSLeu were as follows: R83A, R163A and R203A mutants showed 6.7%, 70% and 44% hydroxylation activity toward NSLeu, compared with the wild-type, respectively (Fig. 5*A*). The Gly79 and Phe261 mutants showed reduced activity (6.2–19%) and the T77V mutant showed 6.4% activity compared with the wild-type. On the other hand, the Arg mutants showed less than 20% hydroxylation activities toward NSPhe compared with the wild-type (Fig. 5*B*). The mutants of Thr77, Gly79 and Phe261 except the T77S one also caused a significantly reduced hydroxylation activity (less than 5%) toward NSPhe compared with the wild-type enzyme. Notably, the T77S mutant retained almost the same activity as the wild-type. The G79A and F261L mutants also had significantly reduced hydroxylation activities (1.3% and 20%, respectively) toward NSVal compared with the wild-type enzyme (Fig. 5*C*). Based on the predicted binding model and the results of mutation analyses, we reconstructed the model of NSLeu and NSPhe in the SadA.Zn(II).α-KG structure (Fig. 5*D*).

## Discussion

In this study, we determined the crystal structures of SadA.Zn(II) and SadA.Zn(II).α-KG. SadA is the first enzyme shown to catalyze the hydroxylation of the *N*-substituted branched-chain and aromatic L-amino acids (data not shown). We also predicted and verified the residues related to substrate recognition around the active site by biochemical analyses. Although we do not obtain the crystal structure of the complex of SadA with an *N*-substituted branched-chain L-amino acid, substrate-binding model analyses combined with the activity assays of various mutants suggest how SadA binds its substrates. SadA showed a different activity toward *N*-substituted branched-chain L-amino acids. Based on the constructed model, we predict that the binding site of the *N*-succinyl group is located in an electropositive-rich cavity by the formation of salt bridges with the side chains of Arg83, Arg163 and Arg203. Consistent with the proposed binding mode of the *N*-succinyl group above, the R83A, R163A and R203A mutants showed reduced hydroxylation activities (Fig. 5*A*). Therefore, SadA shows a high level of activity toward *N*-succinyl branched-chain L-amino acids compared with other *N*-substituted branched-chain L-amino acids (10), which have no additional negatively-charged substituent.

Furthermore, the G79A/V and F261L/A mutants exhibit a significant loss of the hydroxylation activities (Fig. 5*A and 5B*). The hydrophobic interactions of the side chain of *N*-succinyl amino acid probably are formed with the main chain of Gly79 and the phenyl ring of Phe261 (Fig. 5*D*). G79A/V mutants may increase the steric interference and thereby allow the substrates not to enter deeply into the pocket. The decreased activity of F261L/A mutants revealed that the hydrophobic interaction between the side chains of the substrates and the phenyl ring of Phe261 played an important role in substrate recognition. The reduced activity of related mutants is consistent with the proposed mode of substrate binding. It is noteworthy that the T77V mutant exhibited extremely low activity toward two substrates while the T77S mutant showed no decrease in activity compared with the wild-type, indicating that the hydroxy group of Thr77 is important for substrate binding. Thr77 is predicted to bind the carboxyl group of the substrate. In addition, the methyl group of Thr77 is located at the entrance of the substrate-binding pocket (Fig. S3). Because the hydroxylation activity could not be improved by T77S, the methyl group of Thr77 does not contribute to the substrate recognition or the steric interference at the substrate entrance.

In the NSPhe-binding model, NSPhe shared a similar binding mode with NSLeu at the active site (Fig. 5*D*). However, the binding would cause steric hindrance with Gly79 and/or Phe261 because the activity toward NSPhe is lower than that toward NSLeu. F261L/A mutants were considered to relieve the steric hindrance, but they could not improve the activity, suggesting the importance of hydrophobic and/or stacking interactions with NSPhe (Fig. 5*B*). On the other hand, we found that G79A substitution caused the more serious effect of steric hindrance toward NSPhe compared with that toward NSLeu. This observation suggests that the alanine substitution of Gly79 may form a gate to block the entry of NSPhe into the binding site compared with NSLeu.

**Figure 6:**
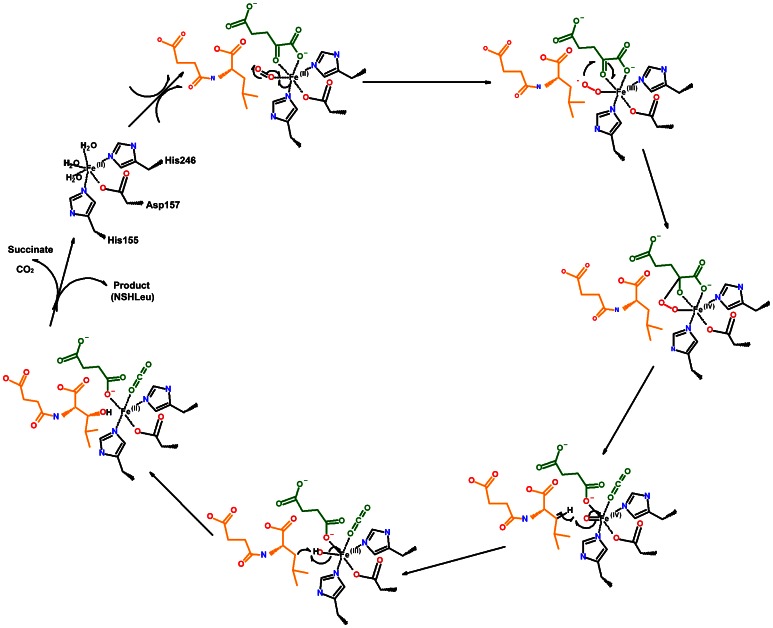
Proposed mechanism of the reaction catalyzed by SadA. The reaction proceeds through a radical mechanism involving an iron-oxo intermediate. Amino acid side chains of the enzyme are colored black, α-KG, succinate and CO_2_ light green, the secondary substrate orange, and the oxygen atoms derived from molecular oxygen (O_2_) red.

Based on the substrate-binding model, it is proposed [6], [13], [27]–[29] that SadA catalyzed the C3-hydroxylation of *N*-substituted branched-chain L-amino acids to produce a chiral molecule using the proposed mechanism (Fig. 6). Briefly, the substrate binds in close proximity to the active site, and the Fe(II) and oxygen can react to generate a Fe(III)-superoxo species, which attacks the 2-ketogroup of α-KG leading to a Fe(IV) = O intermediate. The Fe(IV) = O intermediate then abstracts a hydrogen radical from the C-3 position of the NSLeu substrate. This results in the Fe(III)-OH and the substrate radical being in proximity to each other in the active site. Radical recombination involves OH• transfer to the substrate radical that yields the NSHLeu, and then regenerates SadA to the resting state after the hydroxylated substrate and succinate are released. The key to the catalytic strategy is the generation of the Fe(IV) = O intermediate that removes a hydrogen atom from a carbon site of a bound substrate [30]–[32].

In summary, our structural and biochemical studies provided molecular insights into the SadA hydroxylation reaction mechanism. They reveal the structural basis of the substrate specificity and stereoselective hydroxylation. Further research will focus on the enhancement of hydroxylation activity toward not only *N*-succinyl branched-chain L-amino acids but also NSPhe. Modified SadA will also serve as a model for commercial-scale manufacture of pharmaceuticals in which an enzyme is desired as the target of an industrial biocatalyst.

### Accession Numbers

The atomic coordinate and structure factor (code: 3W20 and 3W21) have been deposited in the Protein Data Bank, Research Collaboratory for Structural Bioinformatics, Rutgers University, New Brunswick, NJ (http://www.rcsb.org).

## Supporting Information

Figure S1 Superdex 200 size-exclusion chromatography of SadA. Ovalbumin (43 kDa) and conalbumin (75 kDa) were used to create the calibration curve (dotted lines). A single peak corresponding to a dimer was observed. The scale at the bottom indicates the elution volume.
(TIF)

Figure S2 2F_0_−F_c_ electron density map of α-KG and Zn(II) contoured at 1.0 sigma. The HXD/EX_n_H motif is shown as white sticks.
(TIF)

Figure S3 Electrostatic surface potential as displayed in blue for positive (5 kTe^−1^), red for negative (−5kTe^−1^) and white for neutral. The black ellipse indicates the predicted substrate-binding pocket. The residues which are related to substrate binding are shown as green sticks.
(TIF)

Table S1 Primer used for the construction of SadA mutants.
(DOC)

Table S2 Metal analysis of SadA.
(DOC)
