# Supplementary figures and images for: Crystal Structure of a Novel N-Substituted L-Amino Acid Dioxygenase from Burkholderia ambifaria AMMD
Source: PLoS One. 2013 May 28;8(5):e63996. doi: 10.1371/journal.pone.0063996 (PMC3665795; doi:10.1371/journal.pone.0063996)

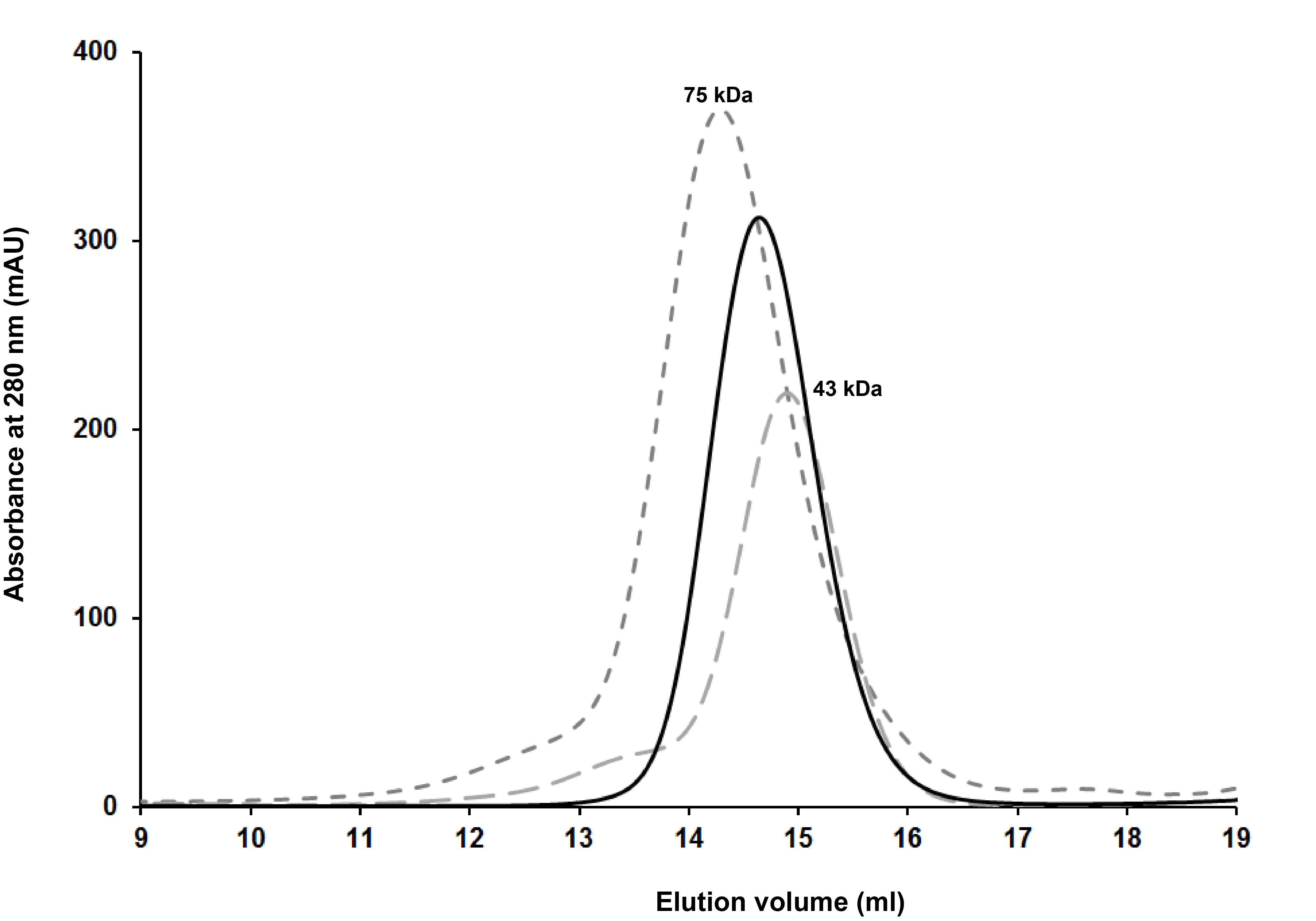

Supplement: Figure S1 — Superdex 200 size-exclusion chromatography of SadA. Ovalbumin (43 kDa) and conalbumin (75 kDa) were used to create the calibration curve (dotted lines). A single peak corresponding to a dimer was observed. The scale at the bottom indicates the elution volume. (TIF) [file pone.0063996.s001.tif]

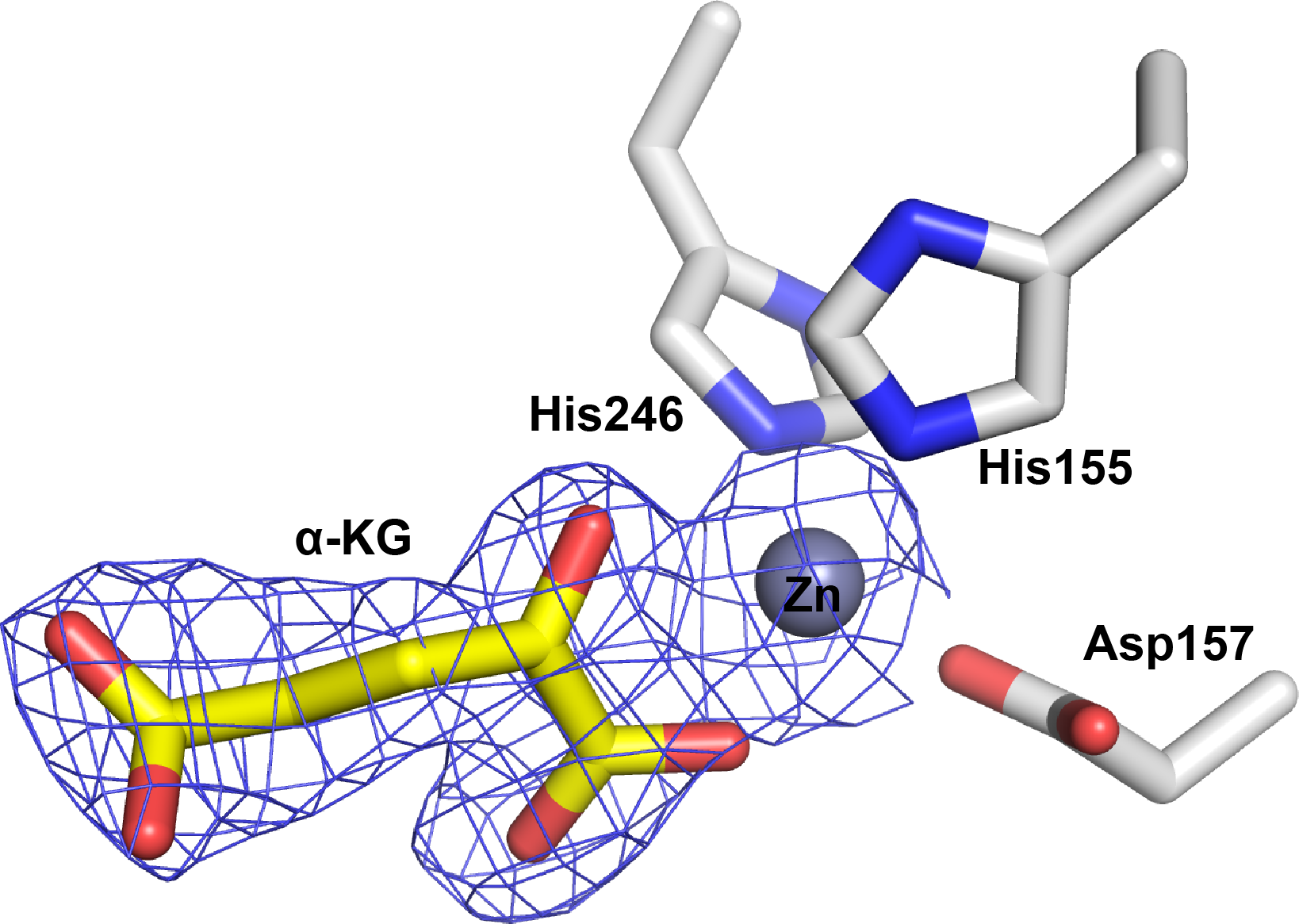

Supplement: Figure S2 — 2F0−Fc electron density map of α-KG and Zn(II) contoured at 1.0 sigma. The HXD/EXnH motif is shown as white sticks. (TIF) [file pone.0063996.s002.tif]

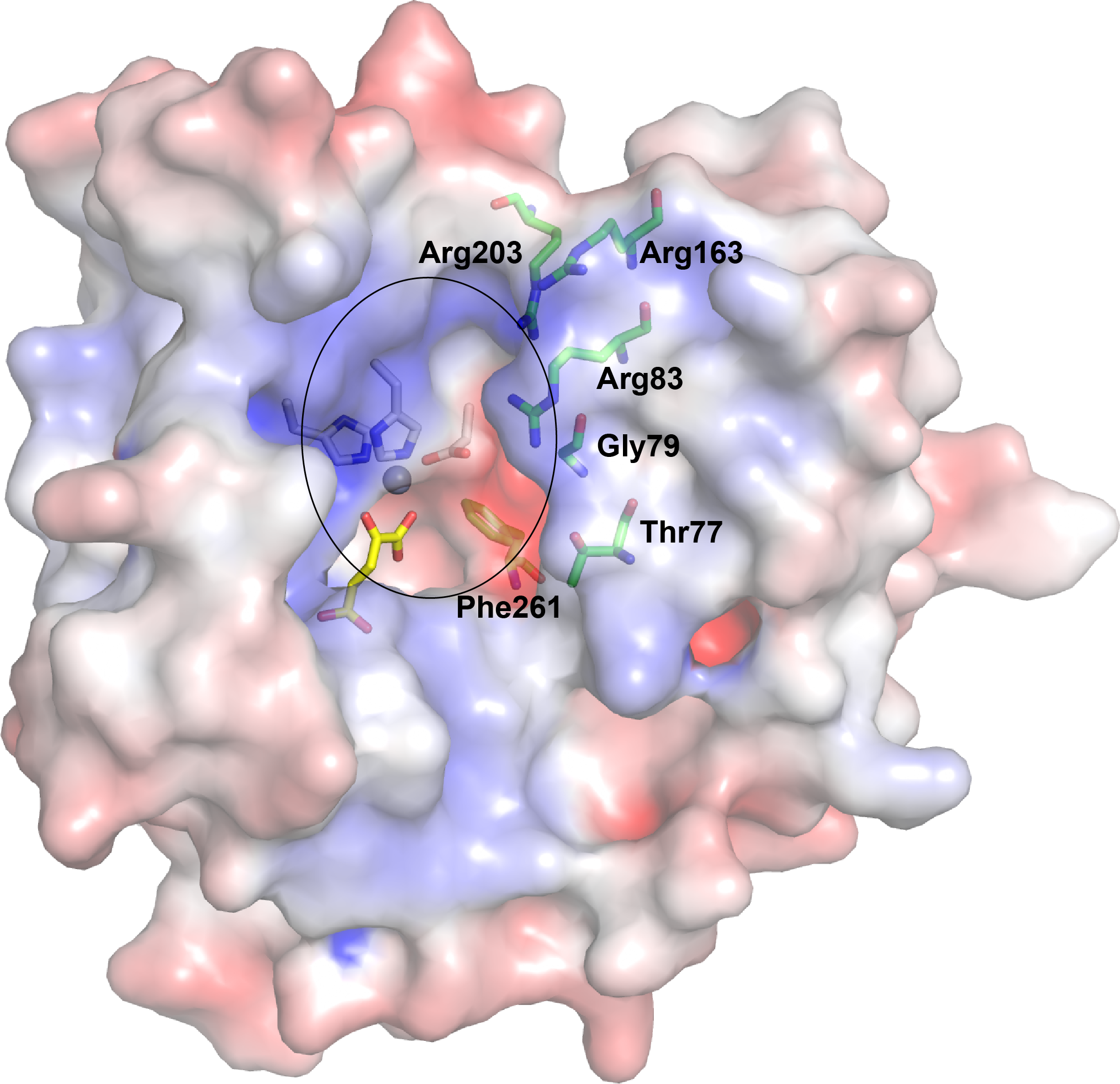

Supplement: Figure S3 — Electrostatic surface potential as displayed in blue for positive (5 kTe−1), red for negative (−5kTe−1) and white for neutral. The black ellipse indicates the predicted substrate-binding pocket. The residues which are related to substrate binding are shown as green sticks. (TIF) [file pone.0063996.s003.tif]
