# Supplementary material for: Crystal Structure of a Novel N-Substituted L-Amino Acid Dioxygenase from Burkholderia ambifaria AMMD
Source: PLoS One. 2013 May 28;8(5):e63996. doi: 10.1371/journal.pone.0063996 (PMC3665795; doi:10.1371/journal.pone.0063996)
Supplement: Table S1 — Primer used for the construction of SadA mutants. (DOC) [file pone.0063996.s004.doc]

**Table S1.** Primer used for the construction of SadA mutants. The bold face letters indicate the mutation generated.

| Primer name | Sequence of primer |
| --- | --- |
| T77V_F | GCCACGTCGGTC**GTG**CTCGGCCAGCTC |
| T77V_R | GAGCTGGCCGAG**CAC**GACCGACGTGGC |
| T77S_F | GCCACGTCGGTC**AGC**CTCGGCCAGCTC |
| T77S_R | GAGCTGGCCGAG**GCT**GACCGACGTGGC |
| G79A_F | GTCGGTCACGCTC**GCG**CAGCTCCAGCGTG |
| G79A_R | CACGCTGGAGCTG**CGC**GAGCGTGACCGAC |
| G79V_F | GTCGGTCACGCTC**GTG**CAGCTCCAGCGTGAAC |
| G79V_R | GTTCACGCTGGAGCTG**CAC**GAGCGTGACCGAC |
| R83A_F | CTCGGCCAGCTCCAG**GCG**GAACAGGGGGAC |
| R83A_R | GTCCCCCTGTTC**CGC**CTGGAGCTGGCCGAG |
| R163A_F | CGTGAGCTACGGG**GCC**GACACCGTGAAC |
| R163A_R | GTTCACGGTGTC**GGC**CCCGTAGCTCACG |
| R203A_F | CCGACCGGAATCC**GCC**GCCGCACTCGAC |
| R203A_R | GTCGAGTGCGGC**GGC**GGATTCCGGTCGG |
| F261L_F | GACGCACGATGGGCCTG**CTG**CTGATCCACACGGAAG |
| F261L_R | CTTCCGTGTGGATCAG**CAG**CAGGCCCATCGTGCGTC |
| F261A_F | GCACGATGGGCCTG**GCG**CTGATCCACACGGAAG |
| F261A_R | CTTCCGTGTGGATCAG**CGC**CAGGCCCATCGTGC |
