# Supplementary material for: Crystal Structure of a Novel N-Substituted L-Amino Acid Dioxygenase from Burkholderia ambifaria AMMD
Source: PLoS One. 2013 May 28;8(5):e63996. doi: 10.1371/journal.pone.0063996 (PMC3665795; doi:10.1371/journal.pone.0063996)
Supplement: Table S2 — Metal analysis of SadA. (DOC) [file pone.0063996.s005.doc]

**Table S2.** Metal analysis of SadA.

|  | Fe | Zn | Mn | Ni |
| --- | --- | --- | --- | --- |
| Metal concentration (ppm) | 0.011 | 0.150 | -0.001 | 0.021 |
| No. of metal ion per subunit | 0.027 | 0.32 | *n.d.* | 0.049 |

*n.d.* not detected

For the identification of the metal ion bound to the active site, we performed inductively coupled plasma atomic emission spectroscopy (ICP-AES) determination. The purified SadA was concentrated to 23 mg ml^-1^ and the buffer was used as blank. The concentration of Zn ion is about 7 to 14-fold higher than those of other metal ions (Fe, Mn, Ni), and number of Zn ion is about 0.32 per subunit of SadA.
